# Knockdown of Serine–Arginine Protein Kinase 3 Impairs Sperm Development in *Spodoptera frugiperda*

**DOI:** 10.3390/insects16121256

**Published:** 2025-12-11

**Authors:** Yilin Song, Yi Zhou, Ruoke Wang, Bing Zhang, Zhongwei Li, Xiangyu Liu, Dandan Li

**Affiliations:** Henan Key Laboratory of Insect Biology, The International Joint Laboratory of Insect Biology in Henan Province, Nanyang Normal University, 1638 Wolong Road, Nanyang 473061, China; 15713813920@163.com (Y.S.); 13943626767@163.com (Y.Z.); 15028842796@163.com (R.W.); zb964034798@163.com (B.Z.); lzw17337796813@163.com (Z.L.); 17516567116@163.com (X.L.)

**Keywords:** *Spodoptera frugiperda*, eupyrene sperm, apyrene sperm, *SRPK3*

## Abstract

Lepidopterans produce two distinct types of sperm: nucleated eupyrene sperm for fertilization and anucleate apyrene sperm for auxiliary functions. However, the mechanisms underlying sperm dimorphism in fall armyworm *Spodoptera frugiperda* remain poorly understood. Serine–Arginine Protein Kinases (SRPKs) are a class of kinases that catalyze the phosphorylation of SR proteins, but recent studies have shown that SRPK is critical for chromatin remodeling of sperm in mammals. Whether SRPK is involved in lepidopteran spermatogenesis is completely unknown. Here, we describe the entire process of elongation and maturation of both eupyrene and apyrene sperm bundles in *S*. *frugiperda*. The eupyrene sperm bundles elongated from the 3-day-old 6th-instar larvae, transiently forming a bowling-pin shape prior to cytoplasmic extrusion and finally maturing into structures with a fan-shaped head and slender tail after eclosion. In contrast, apyrene sperm bundles originated at 2-day-old pupae, where they underwent immediate nuclear extrusion and elongated into bundles that later coiled into a mature, spindle-shaped spool conformation in male adults. Larval knockdown of *Serine–Arginine Protein Kinase 3* (*SRPK3*) significantly reduced apyrene sperm ratio and induced precocious maturation of eupyrene sperm, accompanied by acrosomal malformations. Furthermore, we observed a marked downregulation of cytoskeletal genes—including *α-tubulin* and *cofilin*—in non-testicular tissues and *β-actin* in testicular tissues. In contrast, the expression of *dynamin* and *Lasp* was upregulated in the testis and non-testicular tissues, respectively. Our results indicate that *SRPK3* regulates both apyrene sperm differentiation and eupyrene sperm maturation by modulating the expression of cytoskeletal components, which provides new clues for lepidopteran spermatogenesis.

## Simple Summary

*Spodoptera frugiperda* is a major global pest that causes severe damage to key crops, with its high reproductive capacity being a key driver of its rapid expansion worldwide. However, the mechanisms underlying sperm development in this species remain unclear. In this study, we provide the detailed characterization of the entire elongation and maturation process of both eupyrene and apyrene sperm bundles in *S*. *frugiperda*. We precisely determined the timing of elongation and documented the morphological changes in both sperm types. Furthermore, knockdown of Serine–Arginine Protein Kinase 3 (*SRPK3*) significantly reduced the proportion of apyrene sperm and induced precocious maturation of eupyrene sperm. These changes were accompanied by substantial alterations in the expression of cytoskeletal genes, indicating that *SRPK3* might regulate both apyrene sperm differentiation and eupyrene sperm maturation through modulation of cytoskeletal gene expression. These findings provide new clues for the study of lepidopteran spermatogenesis and pest control.

## 1. Introduction

The fall armyworm, *Spodoptera frugiperda* (J.E. Smith), a lepidopteran pest from the family Noctuidae [1], is a highly destructive transboundary pest that poses a severe threat to global food security. Since 2016, *S*. *frugiperda* has expanded its range to Africa, Asia, and Oceania, where it has become a major invasive pest [2]. This extremely polyphagous insect feeds on over 350 plant species, like maize, rice, and sorghum [3]. The pest’s exceptional capacity for long-distance migration, high reproductive potential, and inherent resistance to many pesticides make it difficult to control. Therefore, elucidating its reproductive mechanisms to develop targeted control strategies represents a promising approach for sustainable management.

Like most lepidopteran insects, male fall armyworms produce two distinct types of sperm during development: nucleated eupyrene sperm and anucleated apyrene sperm [4,5]. Both eupyrene and apyrene sperm are produced in the same testicular follicles. Eupyrene sperm possess highly condensed chromatin within the sperm head and can fertilize the egg [6]. In contrast, apyrene sperm are shorter, with loosely packed chromatin located in the middle of the cell, and form elongated mature bundles after nuclear extrusion [7]. Although apyrene sperm are non-fertilizing, they play an indispensable role in facilitating fertilization. Several hypotheses have been proposed for their function, including sperm competition [8], nutritional support [7,9], and the provision of kinetic energy for eupyrene sperm [10,11].

Spermatogenesis is a dynamic process encompassing the proliferation and differentiation of spermatogonia, meiosis of spermatocytes, and spermiogenesis [12]. The mechanism underlying lepidopteran sperm dimorphism has been extensively studied in the silkworm *Bombyx mori* [4,13,14,15], from which both eupyrene and apyrene sperm are produced in the same testicular follicles. Spermatogonia undergo mitotic divisions, forming 64 spherical spermatogonial cysts. Each cyst then undergoes two successive meiotic divisions, resulting in 256 spermatids. Then, these spermatids subsequently enter the elongation phase [16]. The elongation process of spermatids shows considerable similarity between Drosophila and silkworms. In the early stage of elongation, the nuclei become positioned at one end of the cyst, while the developing sperm tails localize to the opposite end. The spermatids elongate overall, adopting a filamentous morphology, and remain interconnected within the cyst by cytoplasmic bridges. As elongation proceeds, the chromatin undergoes progressive condensation, and the nuclear shape transitions from spherical to a compact, needle-like form. Meanwhile, the acrosome, derived from the Golgi apparatus, elongates along with the nucleus. The centriole gives rise to the flagellar axoneme, which extends progressively. Additionally, two paracrystalline structures of unequal size, formed by mitochondrial fusion, envelop the axoneme and constitute the sperm tail [17]. Upon completion of spermatid elongation, sperm bundles within the cyst must undergo individualization to form motile, mature spermatozoa. This process relies on multiple actin-associated proteins, including Shibire (Shi), Dynamin/Jagular (Jar), Dynamitin (Dmn), and Lasp, which localize to actin cones and are essential for cone assembly around nuclei and cone stability [18,19,20,21]. Other proteins involved in actin-based movement—such as Salto, Chickadee (Chic), the dynein–dynactin complex, and ubiquitin-specific protease 14 (Usp14)—also participate in this process [22,23,24,25,26]. Disruption of the dynein–dynactin complex impairs synchronized cone movement toward the spermatid tail [22,23].

Furthermore, during spermiogenesis, histones are replaced by protamines to achieve highly condensed packaging of paternal DNA within the sperm head [27,28]. Following fertilization, the paternal genome undergoes a programmed reversal of this histone-to-protamine exchange, leading to chromatin decondensation to facilitate the fusion of parental chromosomes [29,30]. Gou et al. demonstrated that maternally derived Serine–Arginine Protein Kinase 1 (SRPK1) catalyzes the phosphorylation of protamine 1 (P1), thereby regulating the initiation of paternal chromatin remodeling in mouse zygotes [31].

In Lepidoptera, eupyrene sperm exhibit highly condensed chromosomes, whereas those in apyrene sperm are loosely packed; whether SRPK plays a role in this intricate sperm dimorphism remains unknown. SRPKs are a major class of kinases that specifically catalyze the phosphorylation of SR proteins [32]. These kinases regulate critical cellular processes such as pre-mRNA alternative splicing [32,33], cell division, and differentiation [15,34]. Mammals possess three SRPK subfamilies: SRPK1, SRPK2, and SRPK3. Among them, SRPK1 and SRPK2 have been widely studied as regulators of alternative splicing and mRNA maturation [35,36,37], transduction of growth signaling [38], chromatin reorganization [39], cell cycle [32] and metabolic signaling [40], whereas SRPK3 exhibits tissue-specific expression, being predominantly present in heart and skeletal muscle from embryogenesis through adulthood [36,41,42,43]. Its expression is controlled by a muscle-specific enhancer regulated by *mef2*, and it contributes to tissue-specific alternative splicing in muscle cells [44]. Functional roles of SRPKs participate in sperm chromatin remodeling, indicating they might play a role in the sperm development of Lepidoptera.

Despite its status as a major invasive agricultural pest, the sperm development of the fall armyworm *S*. *frugiperda* has been poorly studied. Qian et al. found that *SPSL1* is essential for spermatophore formation and sperm activation, and Sun et al. found that *β-tubulin* regulates the development and migration of eupyrene sperm in *Spodoptera frugiperda* [45,46], but the developmental timeline of sperm and regulatory mechanisms underlying sperm dimorphism remain largely uncharacterized.

Here, using fluorescence in situ hybridization (FISH), we characterized the entire process of elongation and maturation of both eupyrene and apyrene sperm bundles in *S*. *frugiperda* and preliminarily elucidated the functional role of *SRPK3* in sperm dimorphism, which provides new clues for the study of lepidopteran spermatogenesis and pest control.

## 2. Materials and Methods

### 2.1. Insect Rearing and Maintenance

A strain of *S*. *frugiperda* (J.E. Smith) was obtained from Keyun Bio. Ltd. (Jiaozuo, Henan, China) and was maintained in our laboratory for over ten generations under controlled conditions of 28 ± 1 °C, 60% ± 10% relative humidity, and a 14:10 h (L:D) photoperiod. Larvae were fed an artificial diet specific to *S*. *frugiperda*, purchased from Keyun Bio. Ltd. (https://3.cn/-2dJbK2H (accessed on 5 March 2022)). After reaching the third instar, larvae were individually placed in labeled containers. Adults were provided with a 10% honey solution.

### 2.2. Detection of Sperm Bundles by Fluorescence in Situ Hybridization (FISH)

FISH was performed to detect the entire process of elongation and maturation of both eupyrene and apyrene sperm in fall armyworm. Testes were dissected from males at developmental stages ranging from 1-day-old sixth-instar larvae to virgin adults. Sperm cells were gently released into 50 μL of phosphate-buffered saline (PBS; 137 mM NaCl, 2.7 mM KCl, 10 mM Na_2_HPO_4_, 1.8 mM KH_2_PO_4_, pH 7.4) and fixed in 4% paraformaldehyde for 10 min. After three washes with PBS, the cells were permeabilized with 1% Triton X-100 for 20 min and washed three more times with PBS. To minimize nonspecific binding, the samples were blocked with 2% bovine serum albumin (BSA) for 30 min and washed three times with PBS. Subsequently, the samples were incubated for 3 h at room temperature with a polyclonal rabbit anti-tubulin primary antibody (Thermo Fisher Scientific, Waltham, MA, USA; diluted 1:500 in 1% BSA). Following three PBS washes, the slides were incubated for 1 h at room temperature with 200 μL of FITC-conjugated goat anti-rabbit IgG (Thermo Fisher Scientific, Waltham, MA, USA; diluted 1:500 in 1% BSA), followed by a final series of three PBS washes. Finally, 150 μL of DAPI staining solution was added, and the slides were incubated in the dark for 10 min. The prepared slides were mounted in glycerol and visualized using a fluorescence microscope (Zeiss, Oberkochen, Germany). The experiment was conducted with three replicates per group, each comprising 5 male individuals. We started with twice the number of larvae needed for sampling to ensure an adequate supply of males. The insects were then sexed based on the dissection of testes and ovaries.

### 2.3. dsRNA Synthesis for RNAi

Total RNA was isolated from the whole body of fourth-instar larvae of *S*. *frugiperda* (using non-sexed individuals, with midgut content removed) using TRIzol reagent (Thermo Fisher, Waltham, MA, USA). First-strand cDNA was then synthesized using the PrimeScript™ II 1st Strand cDNA Synthesis Kit (TaKaRa, Dalian, China). The *SRPK3* gene (XM_035588961.2) was amplified through PCR with the primers listed in Table 1. Double-strand RNA (dsRNA) targeting *SRPK3* was synthesized employing the T7 RiboMAX™ Express RNAi System (Promega, Madison, WI, USA). The concentration of the synthesized dsRNA was quantified using a NanoDrop 2000 spectrophotometer (Thermo Scientific, Waltham, MA, USA).

### 2.4. dsRNA Microinjection Through Hemolymph

To knock down *SRPK3*, microinjections were performed on the hemolymph of 2-day-old pre-pupae of *S*. *frugiperda* (5 µg of dsRNA each) using a PL-100A microinjector (Eppendorf, Hamburg, Germany). Pupae were subsequently housed in a rearing chamber under standard conditions for continuous observation of phenotypic alterations and survival. A control group was established by injecting dsRNA targeting the *eGFP* gene under identical conditions. The experiment was conducted with three replicates per group, each comprising 15 individuals. We started with twice the number of larvae needed for sampling to ensure an adequate supply of males. The pre-pupae and pupae were then sexed based on the method described by Dong and Liu et al. (2019, 2024) [47,48].

### 2.5. Detection of Sperm Morphological Changes After SRPK3 Knockdown

Testes were dissected daily from the 1-day-old pupae to adults (with 7 days for pupae and 1 day for adults). Sperm cells were isolated and fluorescence-stained with the method above. The experiment was conducted with three replicates, each comprising 5 individuals. A total of 300 sperm bundles were counted, and the proportion of apyrene sperm bundles was calculated. A control group was established by injecting dsRNA targeting the *eGFP* gene under identical conditions.

### 2.6. Quantitative Real-Time PCR for Gene Expression Analysis

Testes and non-testes tissues (referring to the remaining tissues with testes and midgut contents removed) were dissected from male pupae at 48 h post-injection of dsRNA-*SRPK3* and dsRNA-*eGFP*. Total RNA was extracted using the TRIzol method. First-strand cDNA was synthesized from 2 μg of total RNA using oligo d(T)_15_ primers and the PrimeScript™ II 1st Strand cDNA Synthesis Kit (TaKaRa, Dalian, China). Gene expression levels were analyzed by quantitative real-time PCR (qRT-PCR) using FS Universal SYBR Green Master mix (Roche, Cornwall, UK) on a CFX96™ Real-Time PCR Detection System (Bio-Rad, Hercules, CA, USA). The thermal cycling protocol consisted of an initial denaturation at 95 °C for 3 min, followed by 40 cycles of 95 °C for 30 s, 55 °C for 30 s, and extension at 72 °C for 30 s. The primers used are listed in Table 1. The *actin* gene (Accession No. EU100017.1) served as an internal reference for normalization of *SRPK3*. *EF1α* gene (Accession No. PV705599.1) served as an internal reference for normalization of cytoskeletal protein genes, which include *Cofilin* (Accession No. JX087452.1), *Lasp* (Accession No. XM_050702629.1), *Dynamin* (Accession No. XM_035574929.2), *β-actin* (Accession No. OK319028.1) and *α-tubulin* (Accession No. HQ008728.1). Relative mRNA expression levels were calculated using the 2^–ΔΔCt^ method [49], where ΔCt = Ct(target) − Ct(reference) and ΔΔCt = ΔCt(sample) − ΔCt(control). The data represent three independent biological replicates, with each sample measured in technical triplicate. Differences between samples were analyzed using one-way ANOVA and *t*-tests.

### 2.7. Data Analysis

Data analysis was conducted using SPSS Statistics 26.0 for statistical tests and GraphPad Prism 8.0.2 for data visualization. Statistical significance (*p* < 0.05) in qRT-PCR and ratio of apyrene sperm measurements was assessed using Student’s *t*-tests for parametric data or Mann–Whitney U tests for nonparametric data, based on the Shapiro–Wilk test for normality. For multiple comparison analyses, the Bonferroni correction was applied to control for Type I errors.

## 3. Results

### 3.1. Elongation and Maturation of Eupyrene Sperm Bundles in S. frugiperda

**Figure 1:**
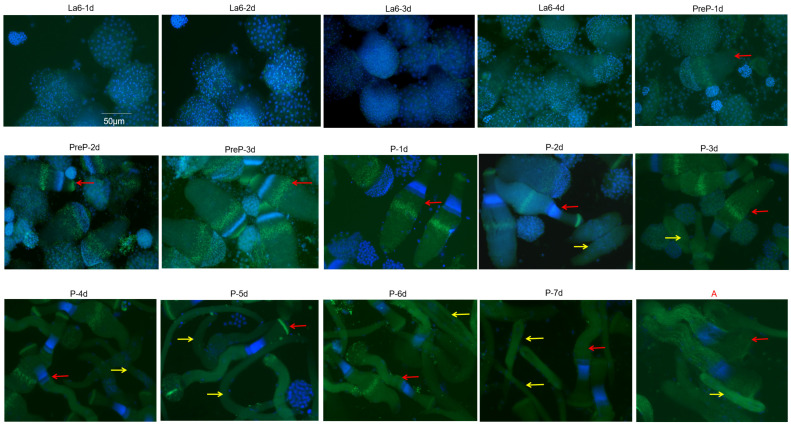
Elongation and maturation of eupyrene and apyrene sperm in *S. frugiperda*. Larval stages (La-1d to La-4d) correspond to 1- to 4-day-old sixth-instar larvae. Pre-pupal stages (PreP-1d and PreP-2d) designate 1- and 2-day-old pre-pupae. Pupal stages (P-1d to P-7d) represent 1- to 7-day-old pupae. The adult stage (A) is a newly enclosed male. Eupyrene sperm bundles are indicated by red arrows, and apyrene sperm bundles by yellow arrows. Scale bar is 50 µm.

To investigate the elongation and maturation process of eupyrene sperm bundles in *S*. *frugiperda*, testes were dissected daily from 1-day-old sixth-instar larvae to adult males, and sperm were analyzed using FISH. Our results showed that spherical spermatogonia underwent mitosis, forming 64 spherical spermatogonial cysts; each cyst contained exclusively eupyrene or apyrene spermatocytes, which underwent two successive meiotic divisions to produce nearly 256 spermatids during the sixth-instar larvae (which lasted 4 days, Figure 1). The differentiation of eupyrene sperm from spherical spermatogonia into bundles began in the 3-day-old sixth-instar larvae. Subsequently, during elongation at the 1-day-old pupal stage, the bundles transformed into a “bowling pin” morphology, with chromosomes aggregated at the neck and tubulin distributed throughout the head and body. From the 2-day-old pupal stage, the bundles gradually narrowed, expelling cytoplasm to form a flattened head and an elongated tail, while a distinct bright line of tubulin remained visible in the acrosomal region. As maturation progressed, tubulin in the acrosomal area gradually dispersed, causing the head to assume a fan shape, and the tail shortened progressively, ultimately forming the mature eupyrene sperm bundle in adult males (Figure 1).

### 3.2. Elongation and Maturation of Apyrene Sperm in S. frugiperda

Apyrene sperm differentiated from spherical spermatogonia into bundles later than eupyrene sperm. The elongated apyrene sperm bundles first appeared in 2-day-old pupae (Figure 1), in which chromosomes were loosely organized in the center of a spindle-shaped cell before being progressively extruded to form an elongated, filamentous sperm bundle. As development proceeds to the late pupal and adult stages, a subset of these filamentous bundles coils into a spindle-shaped spool conformation. These spools were abundantly present in the adult testis (Figure 1), suggesting that the distinct morphology of apyrene sperm may be functionally relevant in assisting eupyrene sperm during fertilization.

### 3.3. Knockdown of SRPK3 Impairs the Development of Apyrene Sperm in S. frugiperda

**Figure 2:**
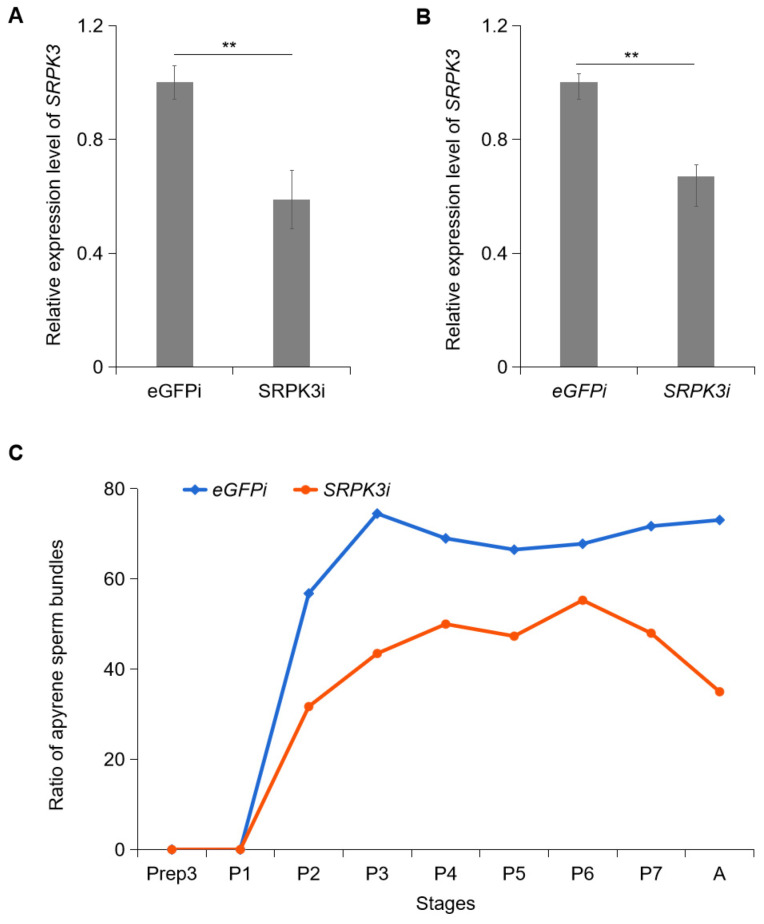
Effects of *SRPK3* knockdown on gene expression and apyrene sperm production. (**A**) *SRPK3* transcript levels in testis (**A**) and non-testis tissue (**B**) after RNAi by qRT-PCR. (**C**) Ratio of apyrene sperm bundles in the total sperm population. Fall armyworms were injected with dsRNA targeting *eGFP* (control, *eGFP*i) or *SRPK3* (*SRPK3*i) at the 2-day-old pre-pupal stage. Samples were collected from subsequent developmental stages: PreP-3d (3-day-old pre-pupae), P-1d to P-7d (1- to 7-day-old pupae), and Adult (A, newly eclosed male). ** *p* ≤ 0.01. The experiment was conducted with three replicates per group, each comprising 15 individuals.

*SRPK1* has been shown to regulate the initiation of early chromatin remodeling in mouse zygotes [31]. To investigate whether *SRPK* plays a role in sperm dimorphism in the fall armyworm, we first identified *SRPK* family genes in *S. frugiperda*. Only one *SRPK* homolog, *SRPK3,* was found. We then injected dsRNA targeting *SRPK3* into the hemolymph of 2-day-old pre-pupae and assessed changes in gene expression by qRT-PCR 48 h post-injection. The results indicated that *SRPK3* expression decreased by 41.22% in testes (Figure 2A, *p* < 0.01) and by 33.17% in other tissues (Figure 2B, *p* < 0.01), compared to the dsRNA-eGFP negative control, respectively. Furthermore, the proportion of apyrene sperm bundles in the total sperm population significantly decreased from the 3-day-old pupal stage (48 h after dsRNA injection) through the adult male stage (Figure 2C).

### 3.4. Morphological Changes in Sperm After SRPK3 Knockdown

**Figure 3:**
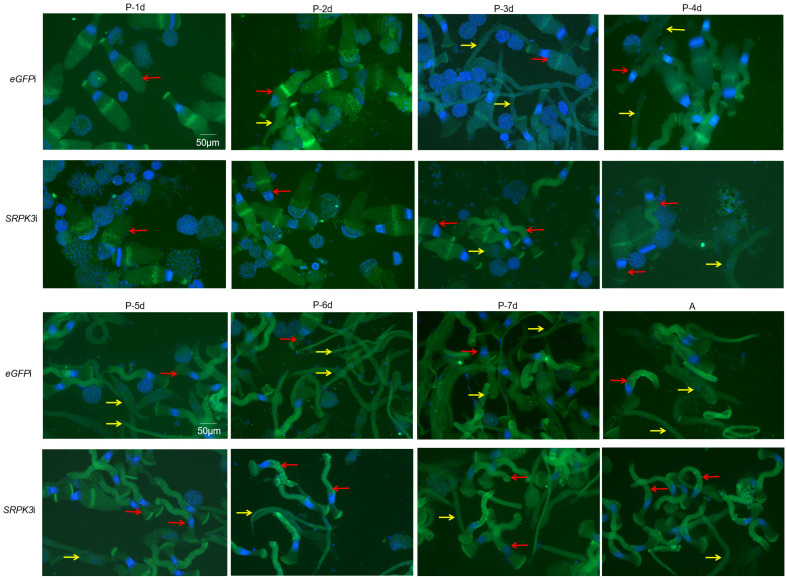
Morphological changes in sperm after *SRPK3* knockdown. Larval stages (La-1d to La-4d) correspond to 1- to 4-day-old sixth-instar larvae. Pre-pupal stages (PreP-1d and PreP-2d) designate 1- and 2-day-old pre-pupae. Pupal stages (P-1d to P-7d) represent 1- to 7-day-old pupae. The adult stage (A) is a newly enclosed male. Eupyrene sperm bundles are indicated by red arrows, and apyrene sperm bundles by yellow arrows. Scale bar is 50 µm.

To further investigate the morphological alterations in sperm development after *SRPK3* knockdown, we utilized fluorescence in situ hybridization (FISH) to assess sperm morphology across multiple developmental stages. The results revealed that *SRPK3* knockdown not only reduced the proportion of apyrene sperm but also decreased the abundance of spindle-spool-shaped apyrene sperm in adults (Figure 3). Interestingly, *SRPK3* knockdown accelerated the maturation of eupyrene sperm, as indicated by the premature appearance of sperm with fan-shaped heads as early as the 6-day pupal stage—a morphology that in controls typically emerges only after eclosion (Figure 3). Meanwhile, the acrosomal structure in a subset of eupyrene sperm appeared flattened and lost the characteristic fan-shaped morphology (Figure 3, P6). These findings imply that *SRPK3* may play a dual role in regulating both apyrene sperm formation and the cytoskeletal organization of eupyrene sperm.

### 3.5. Altered Expression of Cytoskeletal Genes Following SRPK3 Knockdown

**Figure 4:**
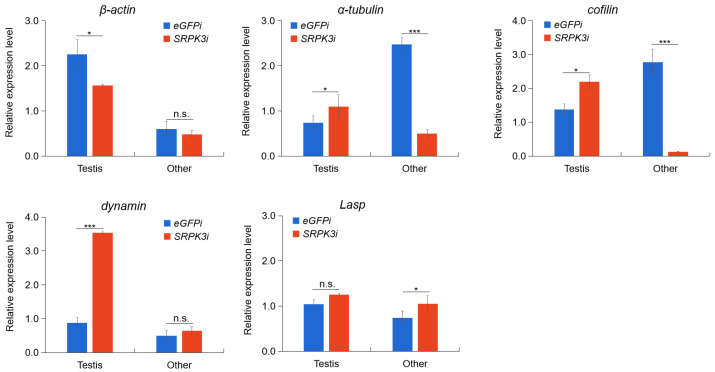
Expression changes in cytoskeletal-related genes following *SRPK3* knockdown. Transcript levels of *β-actin*, *α-tubulin*, *cofilin*, *dynamin*, and *Lasp* were measured by qRT-PCR in *S*. *frugiperda* injected with dsRNA targeting *SRPK3* (*SRPK3*i) or *eGFP* (control, *eGFP*i). “Other” refers to larval tissues after removal of testes and midgut. *EF1α* was used as an internal reference. Data are presented as mean ± SEM from three biologically independent experiments, each with technical triplicates. * *p* ≤ 0.05, *** *p* ≤ 0.001, n.s. (not significant), *p* > 0.05.

To determine whether *SRPK3* regulates the expression of cytoskeletal protein genes, we analyzed the transcript levels of several key factors involved in acrosome formation. Results showed that *α-tubulin* and *cofilin* were significantly downregulated in non-testicular tissues (Figure 4, *p* < 0.001), while *β-actin* was downregulated in the testis (Figure 4, *p* < 0.05). In contrast, expression of *dynamin* was markedly upregulated in the testis (Figure 4, *p* < 0.001), and *Lasp* expression was significantly increased in non-testicular tissues (Figure 4, *p* < 0.05). These results indicate that *SRPK3* is involved in modulating the expression of cytoskeletal components, which may contribute to the observed defects in sperm development and morphology.

## 4. Discussion

Lepidoptera, the second-largest insect order [50], are major agricultural pests. Their high fecundity, which enables multiple generations and massive egg production each year, combined with strong migratory capacity that facilitates long-range dispersal, drives their rapid proliferation. Therefore, an in-depth exploration of the reproductive mechanisms of the fall armyworm and the development of novel control strategies are crucial for significantly mitigating the damage this pest inflicts on crops.

Here, we employed the FISH method to elucidate the whole elongation and maturation process of sperm in *S*. *frugiperda*. Our results indicated that the mitosis of spermatogonia of *S*. *frugiperda* is similar to that in most Lepidoptera [50]. Spherical spermatogonia develop into 64 spherical spermatocyst clusters, which then give rise to approximately 256 spermatids through meiotic divisions during the sixth-instar larval stage. The differentiation of eupyrene sperm from spherical spermatogonia into bundles also resembles that in the silkworm, beginning in the late larval stage—specifically, in 3-day-old sixth-instar larvae. However, the acrosomal region of *S*. *frugiperda* is considerably larger than that of the silkworm.

Regarding apyrene sperm, the characteristic spindle-shaped anucleate sperm with chromosomes loosely packed in the cell center first appeared at the 2-day-old pupal stage. In the silkworm, however, it was observed at the 2-day-old pre-pupal stage, a little earlier than *S*. *frugiperda* [51]. Moreover, mature apyrene sperm bundles coiled into a spindle-shaped spool conformation that is more compact and smaller, contrasting with the filamentous bundles typical of the silkworm [51]. The reproductive strategies of the fall armyworm and the domestic silkworm exhibit marked differences. A single mating pair of *S*. *frugiperda* can produce 1500–2000 eggs [52], far exceeding the silkworm’s 500–600 [53]. This high output is coupled with a batch-laying strategy, an adaptation to unpredictable environments that ensures some offspring survive predation or pesticide application. Moreover, the complex acrosome structure in *S*. *frugiperda* may further contribute to its reproductive success by providing better protection for sperm chromosomes. *Spodoptera frugiperda* seems to have adopted a slower but more robust and competitive pathway to enable it to thrive in challenging environments. In contrast, the silkworm, shaped by domestication, has been optimized for a faster and more synchronized developmental timeline, laying its eggs concentrated within 1–2 days [54].

Several genes have been found to participate in sperm development, such as actin-associated protein genes *Shi*, *Jar*, *Dmn*, and *Lasp* in the process of spermatid elongation and *Salto*, *Chic*, the *dynein–dynactin complex*, and *Usp14* for actin-based movement [18,19,20,21,22,23,24,25,26] in silkworms. A recent study showed that SRPK1 catalyzed the phosphorylation of protamine 1 and regulated the initiation of early chromatin remodeling in mouse zygotes [31], which highlights the importance of SRPK-mediated chromatin relaxation. Here, we found that knockdown of *SRPK3* in fall armyworm significantly reduces the proportion of apyrene sperm and induces precocious maturation of eupyrene sperm, suggesting a potential role for *SRPK3* in chromatin remodeling during apyrene sperm development. Furthermore, the expression of cytoskeletal genes—including *β-actin*, *α-tubulin*, and *cofilin*—was downregulated, and dynamin was upregulated after *SRPK3* knockdown. The altered expression of cytoskeletal genes following *SRPK3* knockdown suggests that *SRPK3* may influence apyrene sperm differentiation by modulating cytoskeletal protein expression. Taken together, these results suggest that *SRPK3* likely affects apyrene sperm differentiation through the regulation of cytoskeletal proteins. Meanwhile, knockdown of *SRPK3* promoted the maintenance of a compact chromatin state, thereby facilitating the maturation of eupyrene sperm. But the direct downstream target genes of *SRPK3* might be other genes like transcription factors, which need much more evidence.

Kinase-mediated phosphorylation is widely recognized as a critical regulatory mechanism in spermatogenesis, influencing germ cell development from spermatogonia to spermatids, as well as subsequent processes including sperm capacitation, motility, the acrosome reaction, and fertilization [55]. The testis-specific serine/threonine kinase (TSSK) family plays essential roles during spermiogenesis [56,57,58]. For example, STK33 phosphorylates multiple proteins linked to infertility and is required for the differentiation of round spermatids into functional sperm. Its loss leads to abnormal manchette structure—tight, straight, and elongated—highlighting its importance in spermatid differentiation and male fertility [59]. These findings suggest that *SRPK3* may similarly phosphorylate proteins associated with fertility, though further evidence is needed to confirm this hypothesis.

## 5. Conclusions

In this study, the entire process of elongation and maturation of both eupyrene and apyrene sperm bundles was elaborated in *S*. *frugiperda*. Meanwhile, we found that knockdown of *SRPK3* decreased cytoskeletal protein expression and significantly reduced apyrene sperm production. It also accelerated the maturation of epyrene sperm in *S*. *frugiperda*. The fall armyworm is a major agricultural pest whose rapid global spread is largely driven by its strong reproductive capacity and migratory behavior. Understanding the regulatory mechanisms of its reproductive development will offer valuable insights and strategies for future pest control.

## Figures and Tables

**Table 1:** Primer sets designed and used in this study.

Genes	Forward Primer (5’-3’)	Reverse Primer (5’-3’)
*SRPK3*-dsRNA (XM_035588961.2)	GAAATTAATACGACTCACTATAGGGCAGGAACCCCTATGTCTGA	GAAATTAATACGACTCACTATAGGCGTCACGGCTATACCCATCT
*eGFP*-dsRNA *(MH070103.1)*	GAAATTAATACGACTCACTATAGGGTACGGCGTGCAGTGCT	GAAATTAATACGACTCACTATAGGGTGATCGCGCTTCTCG
*SRPK3 (XM_035588961.2)*	AAGAAACGGCATAAACTCGG	GCGTATTGCTCGTCCTCATC
*actin (KT218672.1)*	GATGTCGGGACGGGATA	TCATACGGCGAGTGCTT
*Cofilin* (JX087452.1)	ATCAGGGATGAGAAACAAAT	GTACTCGAAGTCGAAGAGGC
*Lasp* (XM_050702629.1)	AGAGGGCCTCCGCTACACTT	GTCGCTGGCTTCGTAATCGT
*Dynamin* (XM_035574929.2)	GGAAAGAGTTCGGTGTTAGA	CTGGTGATATGCCCTTGTTA
*β-actin* (OK319028.1)	CCACCCTGAGTTCTCCAATG	AGTCTCCTGCCAAAGTCCCT
*α-tubulin* (HQ008728.1)	TACGCCCGTGGTCACTACAC	CTCCAGCTTGGACTTCTTGC
*EF1α* (PV705599.1)	ATCGGTGGTATTGGTACGGT	CCTTGGGTGGGTTGTTCTTG

Primers were synthesized by Sangon Biotech (Shanghai, China). The part corresponding to the T7 promoter sequence is underlined.

## Data Availability

The original contributions presented in this study are included in the article. Further inquiries can be directed to the corresponding author.
